# NKG2A and COVID-19: another brick in the wall

**DOI:** 10.1038/s41423-020-0450-7

**Published:** 2020-05-07

**Authors:** Luca Antonioli, Matteo Fornai, Carolina Pellegrini, Corrado Blandizzi

**Affiliations:** 1 Department of Clinical and Experimental Medicine, University of Pisa, 56126 Pisa, Italy; 2 Department of Pharmacy, University of Pisa, 56126 Pisa, Italy

**Keywords:** Viral infection, NK cells

Coronavirus disease 2019 (COVID-19) is a viral infection caused by severe acute respiratory syndrome coronavirus 2 (SARS-CoV-2; formerly designated as 2019-nCoV), a novel betacoronavirus firstly identified during a burst of respiratory illness cases in Wuhan City, Hubei Province, China.^1^ Unfortunately, within a few weeks, the SARS-COV2 virus started to spread globally, producing a pandemic of an extremely spreadable and potentially fatal disease, becoming a cause of great concern for global public health.^1^

Despite the current estimates of COVID-19 case fatality rate suggest that this coronavirus is less deadly than other pathogens driving other large-scale outbreaks, such as SARS, Middle East respiratory syndrome, or Ebola, the main concern is that this infection is able to spread more easily than other diseases, including seasonal influenza.^2^ When considering the virus basic reproduction number (*R*_0_), which is the expected number of cases directly generated by one case in a population where all individuals are susceptible to the infection, a value ranging from 1.4 to 3.9 has been reported for SARS-CoV-2.^3^

From the clinical standpoint, most SARS-CoV-2 infected patients are characterized by mild symptoms including dry cough, sore throat, and fever, and the majority of cases undergo spontaneous regression.^4^ However, some subjects developed various fatal complications, including organ failure, septic shock, pulmonary edema, severe pneumonia, and acute respiratory distress syndrome.^4^ A number of reports called their attention on particular sections of the population, such as elderly, obese, subjects with diabetes or cardiovascular disorders (hypertension, atrial fibrillation, stroke), active cancer, and dementia, in whom COVID-19 has been shown to be more aggressive and often lethal.^4^ By contrast, other sections of the population, such as infants and children, appear to be less prone to infection or develop milder symptoms when infected by SARS-CoV-2.^5^ In parallel, it has been observed also that COVID-19 affects more the males than females.^6^

When stratifying COVID-19 patients by disease severity and crossing these data with the composition of immune cells, an inverse correlation between disease severity and percentage of lymphocytes has been observed.^7^ Indeed, a retrospective study by Tan et al. showed that, at the onset of the disease, severe-cured cases and patients with fatal outcome displayed a reduced percentage of lymphocytes when compared with patients with moderate COVID-19 infection.^7^ Of note, critical patients with lymphocyte percentage <5% over the days following the disease onset were more likely to become critically ill, with need for intensive care therapy and high mortality rate.^7^ By contrast, in patients with moderate infection this parameter displayed very scarce variations after the disease onset, and it was higher than 20% at patient discharge.^7^

Along the same line, Qin et al. described the occurrence of a dysregulated immune response in COVID-19 patients, relating these alterations with the pathological process of SARS-CoV-2 infection.^8^ These authors confirmed a marked decrease in T-cell number, which appeared more pronounced in severe cases.^8^ In addition, they reported that the critical cases were characterized by higher leukocyte counts and neutrophil-to-lymphocyte ratio (NLR), as well as lower percentages of monocytes, eosinophils, and basophils.^8^ No significant differences were noted in the levels of IgA, IgG, and complement proteins C3 or C4 by comparison of mild with severe groups, while IgM decreased slightly in the severe cases.^8^ In parallel, critical patients displayed higher levels of circulating inflammatory cytokines (e.g., IL-2R, IL-6, IL-8, IL-10, and TNF) and infection-related biomarkers (e.g., procalcitonin, serum ferritin, and C-reactive protein) than less severe patients.^8^

A subsequent analysis of lymphocyte subsets allowed to observe that in patients with COVID-19 infection the mean values of the three main lymphocyte populations (T, B, and NK cells) were decreased, and such a decrement was more pronounced in severe cases.^8^ In particular, T and NK cells were markedly below their normal levels, while B cells were within the lower level of their normal range.^8^ By contrast, the percentage of naive T helper cells (CD3^+^CD4^+^CD45RA^+^) increased and memory T helper cells (CD3^+^CD4^+^CD45RO^+^) decreased in severe cases, as compared with less severe cases.^8^ Based on these observations, the authors suggested the surveillance of NLR and changes in the percentages of lymphocyte subsets as useful biomarkers for diagnosis, early screening of critical illness, and driving of treatment.^8^ In particular, high NLR levels, reflecting a worsening of the inflammatory process, seems to be tightly related with a poor prognosis for COVID-19 patients.^9^ Of note this index, emerging as a useful biomarker in several chronic inflammatory disorders and neoplastic diseases, appears to be markedly increased also in the elderly,^10^ diabetic subjects,^11^ hypertension^12^, and obese subjects,^13^ who represent the categories of the population identified as at higher risk of experiencing more serious COVID-19 infection and fatal outcomes.

Recently, Zheng et al. reported that among the lymphocyte populations, CD8^+^ and NK cells, involved mainly in the anti-COVID-19 response, underwent greater alterations in terms of total number and impaired function. In particular, the number of T cells and CD8^+^ T cells was lower in patients with severe disease than in cases with mild disease. Moreover, NK-cell counts were reduced remarkably in severe cases as compared with mild disease patients and healthy controls.^14^ In parallel, besides having a decrease in the numbers of these cells, patients with Covid-19 infection displayed a functional exhaustion of NK and CD8^+^ T cells. Of note, exhausted NK and CD8^+^ T cells showed an increased expression of the CD94/NK group 2 member A (NKG2A) receptor. Interestingly, in patients convalescing after therapy, the number of NK and CD8^+^ T cells was restored, and concomitantly their NKG2A expression was markedly reduced.^14^ These findings allow to hypothesize that the functional exhaustion of cytotoxic lymphocytes associated with COVID-19 infection breaks down the antiviral immunity, and that the enhanced expression of NKG2A, as specifically observed in CD8^+^ and NK cells, could contribute to the maintenance of this blunted antiviral surveillance.

NKG2A is a heterodimeric inhibitory receptor expressed prominently by cytotoxic lymphocytes, such as NK cells and CD8^+^ T cells, that are thereby endowed with the ability of sensing the level of “self” MHC class I on target cells.^15,16^ Indeed, this receptor binds a nonclassical minimally polymorphic HLA class I molecule (HLA-E), which presents peptidic domains derived from leader peptide sequences of other HLA class I molecules, such as HLA-G.^15,16^ Upon binding with peptide-loaded HLA-E, NKG2A transduces inhibitory signals through two inhibitory immune-receptor tyrosine-based inhibition motifs, thus suppressing the cytotoxic activity of these immune cells, and promoting viral spreading during a variety chronic viral infections (e.g., polyoma virus or human cytomegalovirus).^17,18^ Several cytokines, such as IL-6 and IL-10, found to be markedly increased in COVID-19 patients, were shown to elicit an upregulation of NKG2A expression on NK and naive CD8^+^ cells.^19^ In addition, it is noteworthy that IL-6 and IL-8, undergoing an increased release in the early phase of COVID-19 infection, impair the functions of NK cells via STAT3-dependent mechanisms, thus contributing to the reduced cytotoxic capacity of these cells.^20^

**Fig. 1:**
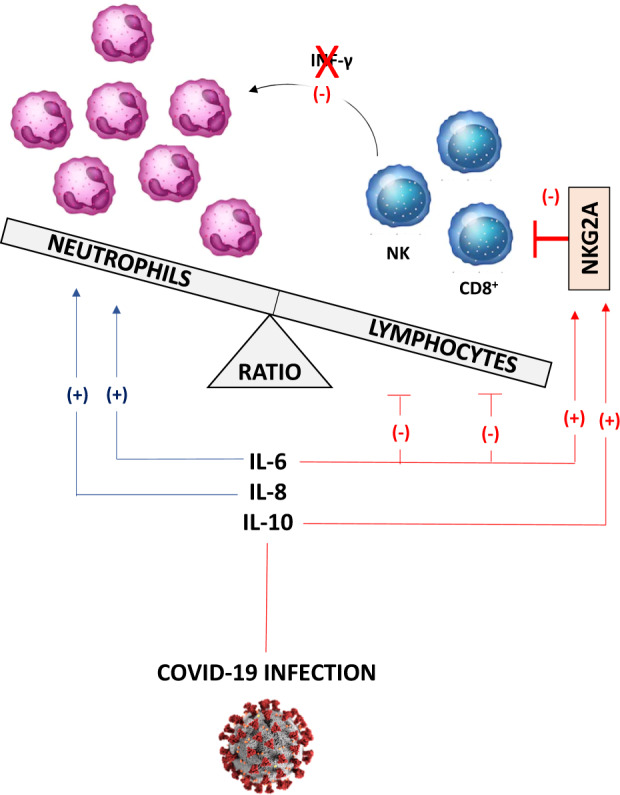
Schematic representation about the role of NKG2A in the alterations occuring between neutrophils and lymphocytes in COVID-19 patients. IL interleukin, NKGD2A CD94/NK group 2 member A

Since it has been widely acknowledged that various leukocytes, including NK and CD8^+^ cells, can be engaged in bidirectional cross-talk with neutrophils, it is conceivable that an alteration of both the number and function of these cells can compromise their mutual equilibrium.^21^ Indeed, it has been well established that contact-dependent interactions, involving neutrophils and NK cells, can stimulate potently the latter cells to produce interferon (IFN)-γ, a versatile cytokine holding a unique position in the antiviral defense system.^21^ Interestingly, IFN-γ inhibits directly the accumulation of pathogenic neutrophils and impairs neutrophil survival in the infected lung, a defense mechanism aimed at counteracting neutrophil accumulation and the related detrimental consequences for the host.^22^ In this context, NKG2A, expressed scarcely on neutrophils, could represent a critical player in the altered balance between neutrophils and lymphocytes occurring typically in COVID-19 patients, thus curbing the expansion of several lymphocyte populations (with particular regard for NK and CD8^+^ cells) without affecting neutrophil expansion (Fig. 1). In parallel, the increased release of cytokines (IL-6 and IL-10) in COVID-19 patients could exacerbate this immune cell disproportion, eliciting an upregulation of NKG2A expression, with a subsequent increment of its inhibitory action on the lymphocyte component, thus compromising the balance between lymphocytes and neutrophils (Fig. 1). In parallel, this scenario could be worsened further by the presence of high IL-6 and IL-8 levels, which can enhance the neutrophil infiltration and activity,^23,24^ while holding an inhibitory action on NK cells^20^(Fig. 1).

At present, there is a strong competition among scientists for the development of vaccines and drugs for the management of COVID-19 pandemic. In parallel, promising and encouraging options are emerging from the field of immunotherapy, which makes use of the components of our immune system to modulate or boost its response. In this regard, based on the above knowledge, monalizumab, a first-in-class humanized IgG4 targeting the NKG2A receptors expressed on cytotoxic NK and CD8^+^ T lymphocytes, might be able to release the brake on these cells, leading to the restoration of an adequate antiviral activity through an increase in the production of IFN-γ.^25^ Of note, the restoration of NK and CD8^+^ cell functions could allow a reorganization of the immune cell pool, which is known to undergo a remarkable unsettlement mainly in the severe cases of COVID-19 infection.

In conclusion, NKG2A appears to represent another brick in the “COVID-19 wall,” owing to its significant involvement in the implementation of adequate immune responses by the host. On this basis, we hypothesize that the anti-NKG2A monoclonal antibody monalizumab, currently under active clinical development for the management of rheumatoid arthritis and several neoplastic disorders,^25^ could represent a viable way for treatment of patients with severe COVID-19 infection, characterized by a sudden and marked reduction of the antiviral activity of NK and CD8^+^ cells.
